# MicroRNA expression profiling of urine exosomes in children with congenital cytomegalovirus infection

**DOI:** 10.1038/s41598-024-56106-1

**Published:** 2024-03-05

**Authors:** Yuka Torii, Takako Suzuki, Yuto Fukuda, Kazunori Haruta, Makoto Yamaguchi, Kazuhiro Horiba, Jun-ichi Kawada, Yoshinori Ito

**Affiliations:** 1 Department of Pediatrics, Nagoya University Graduate School of Medicine, 65 Tsurumai-Cho, Showa-Ku, Nagoya, 466-8550 Japan; 2 Pathogen Genomics Center, National Institute of Infectious Diseases, Toyama 1-23-1, Shinjuku-Ku, Tokyo, 162-8640 Japan; 3 Department of Pediatrics, Aichi Medical University, 1-1 Yazakokarimata, Nagakute, 480-1195 Japan

**Keywords:** Prognostic markers, Viral infection

## Abstract

Congenital cytomegalovirus (cCMV) infection can damage the central nervous system in infants; however, its prognosis cannot be predicted from clinical evaluations at the time of birth. Urinary exosomes can be used to analyze neuronal damage in neuronal diseases. To investigate the extent of neuronal damage in patients with cCMV, exosomal miRNA expression in the urine was investigated in cCMV-infected infants and controls. Microarray analysis of miRNA was performed in a cohort of 30 infants, including 11 symptomatic cCMV (ScCMV), 7 asymptomatic cCMV (AScCMV), and one late-onset ScCMV cases, and 11 healthy controls (HC). Hierarchical clustering analysis revealed the distinct expression profile of ScCMV. The patient with late-onset ScCMV was grouped into the ScCMV cluster. Pathway enrichment analysis of the target mRNAs differed significantly between the ScCMV and HC groups; this analysis also revealed that pathways related to brain development were linked to upregulated pathways. Six miRNAs that significantly different between groups (ScCMV vs. HC and ScCMV vs. AScCMV) were selected for digital PCR in another cohort for further validation. Although these six miRNAs seemed insufficient for predicting ScCMV, expression profiles of urine exosomal miRNAs can reveal neurological damage in patients with ScCMV compared to those with AcCMV or healthy infants.

## Introduction

Cytomegalovirus (CMV) is a pervasive virus. Many people infected with CMV show no significant symptoms. However, fetuses may be affected by CMV infection or reactivation in their mothers. Most congenital CMV infections (cCMV) occur without any symptoms (asymptomatic cCMV; AScCMV), whereas 10–15% of infants with cCMV show physical or laboratory symptoms (symptomatic cCMV; ScCMV)^1^. The representative clinical findings of ScCMV are petechiae, jaundice, hepatosplenomegaly, small gestational age, microcephaly, sensorineural hearing loss (SNHL), and retinal choroiditis. SNHL and neurodevelopmental disabilities mostly affect disease prognosis. Neurodevelopmental disabilities and SNHL may be observed after several months or even years^2^. Because clinical assessments, including neuroimaging, are insufficient to predict neurological prognosis, investigations to reveal neurological damage are needed. With regard to the treatment of ScCMV with apparent neurological abnormalities, six months of oral valganciclovir treatment has shown favorable outcomes, especially in SNHL and neurodevelopmental disabilities^3^, and is currently becoming a common practice. If neurological damage is precisely assessed in patients with cCMV, the indications for valganciclovir treatment might be expanded, and the prognosis of the disease would be improved.

Exosomes are lipid vesicles secreted from cells into body fluids, including the blood, cerebrospinal fluid (CSF), and urine. Exosomes can be transferred across cells and facilitate intercellular communication by transporting their contents, such as microRNAs (miRNAs)^4^. These miRNAs are small non-coding RNAs containing 21–25 nucleotides whose sequences are complementary to mRNAs. The miRNAs are associated with various biological processes, including cell proliferation and differentiation, organ development, and inflammation. Several reports have identified miRNA signatures in many diseases, especially cancers^5^, and several miRNAs have been proven to be distinguishable biomarkers in several cancers^6^. Such miRNAs have also been investigated for central nervous system diseases^7,8^, and they are stable and can circulate throughout the body, which makes it possible to detect disease-specific exosomes in the CSF, blood, and urine^9^. Exosomes can cross the blood–brain barrier bidirectionally^10^, and a growing number of reports have shown a relationship between neurological disorders and peripheral exosomes. Although most of these studies have investigated blood exosomes, urine exosomes have also been analyzed in some studies^11,12^. Urine samples have the advantage that urine collection is usually non-invasive, and the amount of sample required for the assay is small. These are especially important in research on infants.

In this study, urine exosomal miRNAs were analyzed in a cohort of healthy controls, patients with asymptomatic cCMV, and patients with symptomatic cCMV, including late-onset ScCMV, to investigate their potential as biomarkers for assessing neurological damage.

## Results

### Patient characteristics

**Figure 1:**
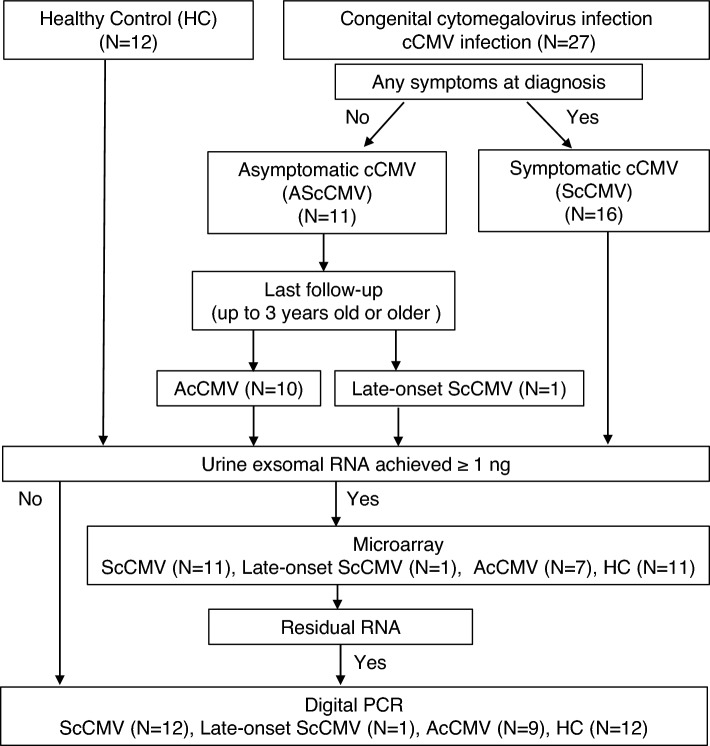
Study design. A total of 39 babies were included in the study. Twelve infants who tested negative for CMV via PCR were included as healthy controls. Twenty-nine samples from the study participants reached sufficient levels of extracted RNA and were subjected to miRNA microarray analysis. Thirty-three samples were subjected to digital PCR for further validation. scCMV, symptomatic congenital cytomegalovirus (CMV) infection; AScCMV, asymptomatic CMV infection; HC, healthy controls.

The study design is summarized in Fig. 1. Twenty-seven babies with cCMV were recruited at Nagoya University for assessment, treatment, and follow-up. They were diagnosed with positive urine CMV PCR results performed within 3 weeks of age. The babies had undergone urine CMV PCR when they were suspected to have cCMV from their mothers’ serostatus (e.g., CMV IgG seroconversion or elevated CMV IgM during pregnancy) or via clinical findings. Sixteen infants were diagnosed with symptomatic cCMV infections (ScCMV). All infants with ScCMV received valganciclovir treatment except one because the patient had exceeded the appropriate age at the first visit. All urine samples were collected before the beginning of valganciclovir treatment. The duration of the treatment varied between 6 weeks and 6 months depending on the period. Eleven babies had no apparent physical signs at the first visit but underwent further laboratory tests, magnetic resonance imaging (MRI), auditory brainstem response, and ophthalmological evaluation. All babies were confirmed to be asymptomatic and were followed up for auditory and neurodevelopmental assessments up to 3 years of age. Of these babies, neurodevelopmental delay became more evident later in one patient (late-onset ScCMV). The neurologist realized the developmental delay in this patient at the 1-year-old visit. The developmental quotient was 73 at the 18-month-old visit. The remaining ten babies remained asymptomatic (AScCMV; asymptomatic cCMV infection). The patient characteristics and the results of the microarray analysis and digital PCR assays are summarized in Tables 1, 2, and 3, respectively.

**Table 1:** Characteristics of patients in the miRNA microarray cohort.

	ScCMV (N = 11)	AScCMV (N = 7)		Late-onset ScCMV (N = 1)	p-value (ScCMV vs. AScCMV)
Gestational age (weeks)	38.9 (31.4–40.1)	39.9 (38.6–40.3)		36	0.111
Birth weight (grams)	2570 (926–3366)	2875 (2498–3526)		2600	0.133
Blood CMV DNA load at 1st visit (IU/mL)	1.1 × 10^4^ (0–1.2 × 10^5^)	1.8 × 10^3^ (0–3.2 × 10^4^)		1.5 × 10^5^	0.081
Urine CMV DNA load at 1st visit (IU/mL)	2.4 × 10^7^ (1.3 × 10^3^–1.1 × 10^8^)	2.7 × 10^6^ (6.5 × 10^5^–3.4 × 10^6^)		5.8 × 10^6^	0.015
Age at sample collection (days)	23 (2–73)	18 (0–88)		127	0.84

*scCMV* symptomatic congenital cytomegalovirus (CMV) infection, *AScCMV* asymptomatic CMV infection.

**Table 2:** Characteristics of patients in the digital PCR cohort.

	ScCMV (N = 12)	AScCMV (N = 9)	Late-onset ScCMV (N = 1)	p-value (ScCMV vs. AScCMV)
Gestational age (weeks)	38.5 (32.3–40.3)	39.6 (38.1–40.3)	36	0.036
Birth weight (grams)	2668 (1602–3366)	2814 (2498–3526)	2600	0.138
Blood CMV DNA load at 1st visit (IU/mL)	4.3 × 10^4^ (0–2.7 × 10^5^)	2.5 × 10^3^ (0–5.3 × 10^4^)	1.5 × 10^5^	0.048
Urine CMV DNA load at 1st visit (IU/mL)	2.0 × 10^7^ (1.3 × 10^3^–1.3 × 10^8^)	3.4 × 10^6^ (6.5 × 10^5^–4.2 × 10^8^)	5.8 × 10^6^	0.757
Age at sample collection (days)	11 (0–35)	21 (0–88)	127	0.127

*scCMV* symptomatic congenital cytomegalovirus (CMV) infection, *AScCMV* asymptomatic CMV infection.

The gestational age and birth weight of a patient with late-onset ScCMV are shown via approximation.

**Table 3:** Clinical findings of the patients with symptomatic congenital cytomegalovirus infection.

	ScCMV		Late-onset ScCMV (N = 1)
	Microarray (N = 11)	Digital PCR (N = 12)	
First visit			
Examination findings			
Petechiae	2 (18.2%)	2 (16.7%)	0 (0%)
Hepatosplenomegaly	1 (9.1%)	2 (16.7%)	
Jaundice	0 (0%)	0 (0%)	
SGA	1 (9.1%)	2 (16.7%)	
Microcephaly	0 (0%)	0 (0%)	
Laboratory findings			
Cytopenia§	2 (18.2%)	4 (33.3%)	
Liver Disfunction*	2 (18.2%)	2 (16.7%)	
Abnormal MRI findings	5 (45.5%)	7 (58.3%)	
Abnormal audiologic findings	7 (63.6%)	5 (41.7%)	
3-year-old visit			
Hearing outcome			
Severe (bilateral or lateral)	5 (45.5%)	3 (25.0%)	0 (0%)
Mild (bilateral or lateral)	1 (9.1%)	2 (16.7%)	0 (0%)
None	5 (45.5%)	7 (58.3%)	1 (100%)
Neurodevelopmental outcome			
Neurodevelopmental delay	4 (36.4%)	3 (25.0%)	1 (100%)
Neurodevelopmental disorder	1 (9.1%)	2 (16.7%)	0 (0%)

*SGA* small for gestational age.

^§^Cytopenia includes any of the following: white blood cell count < 5000/mm^3^, hemoglobin < 12 g/dL, or platelet count < 1.0 × 10^5^/mm^3^.

*Liver dysfunction includes any of the following: ALT level > 100 IU/L or direct bilirubin level > 2 mg/dL.

^¶^Hearing outcomes: severe; cochlear implant, mild; acoustic aid only.

### Western blot analysis

**Figure 2:**
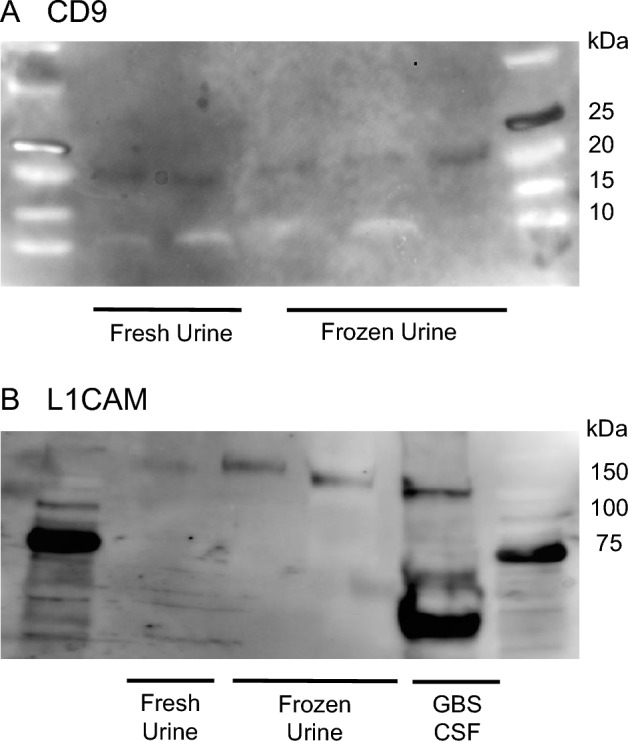
Western blot results of urine samples for exosome analysis. Expression of CD9 in exosomes obtained from frozen and fresh urine samples (**A**). Expression of L1CAM in exosomes from frozen urine, fresh urine (cCMV), and the cerebrospinal fluid of patients with Group B streptococcus infection (**B**).

Prior to exosomal miRNA analysis, the ability to derive urine exosomes was examined by western blotting. CD9, a glycoprotein present on the surface of exosomes, was detected in both fresh and preserved urine exosome samples. L1CAM, a marker for neuron-derived exosomes, was also detected in the urine exosome samples (Fig. 2, Supplementary Figure S1).

### Urine exosomal miRNA microarray analysis

**Figure 3:**
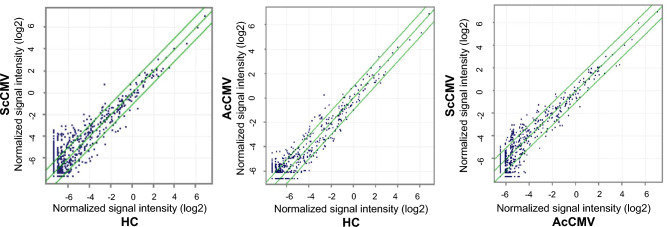
Scatter plot of the miRNA microarray data. Data from the miRNA microarray were graphed on a scatter plot to visualize variations in gene expression between groups. The values on the X- and Y-axes of the scatter plot are the normalized signal values for the samples (log2-scaled). The green lines represent the fold-change (the fold-change value was 2.0).

Exosomal miRNA in the Urine samples collected at the first visit was analyzed. For ScCMV patients, all urine samples were collected before the beginning of valganciclovir treatment. A total of 2549 miRNAs were detected in 30 baby samples. Principal component analysis revealed a subtle separation between the groups (Supplemental Figure S2). The scatter plot in Fig. 3 shows the difference in the expression of each miRNA between the groups. Differentially expressed miRNAs were more noticeable in the ScCMV vs. HC comparison set than in the other pairs (ScCMV vs. AScCMV and AScCMV vs. HC).

**Figure 4:**
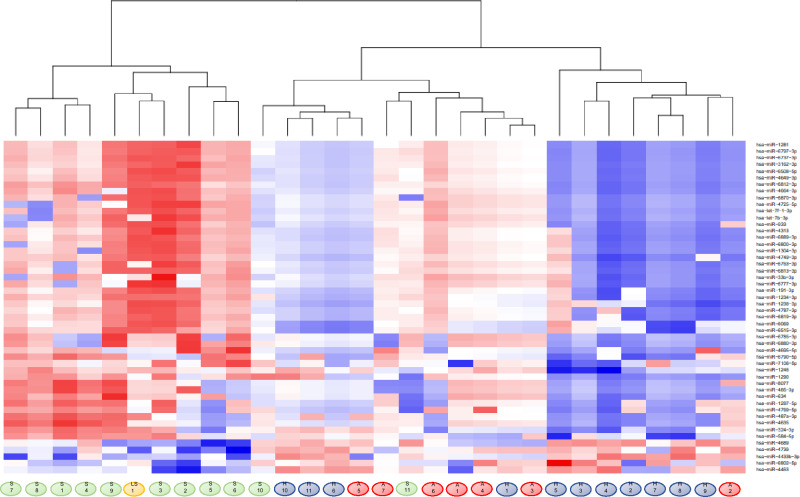
Hierarchical clustering analysis and heatmap of the top-50 significantly differentially expressed miRNAs in patients with ScCMV vs. HC. Red, upregulated; Blue, downregulated. Samples were assigned in each group color under the map. Green, ScCMV; orange, late-onset; red, AScCMV; blue, HC). scCMV, symptomatic congenital cytomegalovirus (CMV) infection; AScCMV, asymptomatic CMV infection; HC, healthy controls.

Differences in miRNA expression levels were compared between groups. Comparative analysis revealed that 71 miRNAs were significantly differentially expressed (false discovery rate, adjusted p-value < 0.05) in patients with ScCMV compared to HC (Supplementary Table S1). No miRNAs were significantly differentially expressed between the two other comparison sets (ScCMV vs. AScCMV and AScCMV vs. HC). The top-50 significantly differentially expressed miRNAs screened by comparing the ScCMV and HC groups were used for the hierarchical analysis (Fig. 4). The patients and controls were divided into four groups. Patients with ScCMVs were grouped into the same cluster. Patients with AScCMV infection were indistinguishable from the controls. Interestingly, patients with late-onset ScCMV were grouped in the same cluster as patients with ScCMV, even though they were asymptomatic when their urine samples were collected.

We further analyzed the relationship between miRNA expression and the severity of sequelae or the viral load of urine at sampling. No relationship between miRNA expression and severity nor viral load was found. To find organ specific miRNA, subanalyses were implemented between ScCMV (SNHL only) vs. AScCMV and between ScCMV (neurodevelopmental sequelae only) vs. AScCMV. No significantly different miRNA was found in the two subanalyses.

### Gene set enrichment analysis

**Figure 5:**
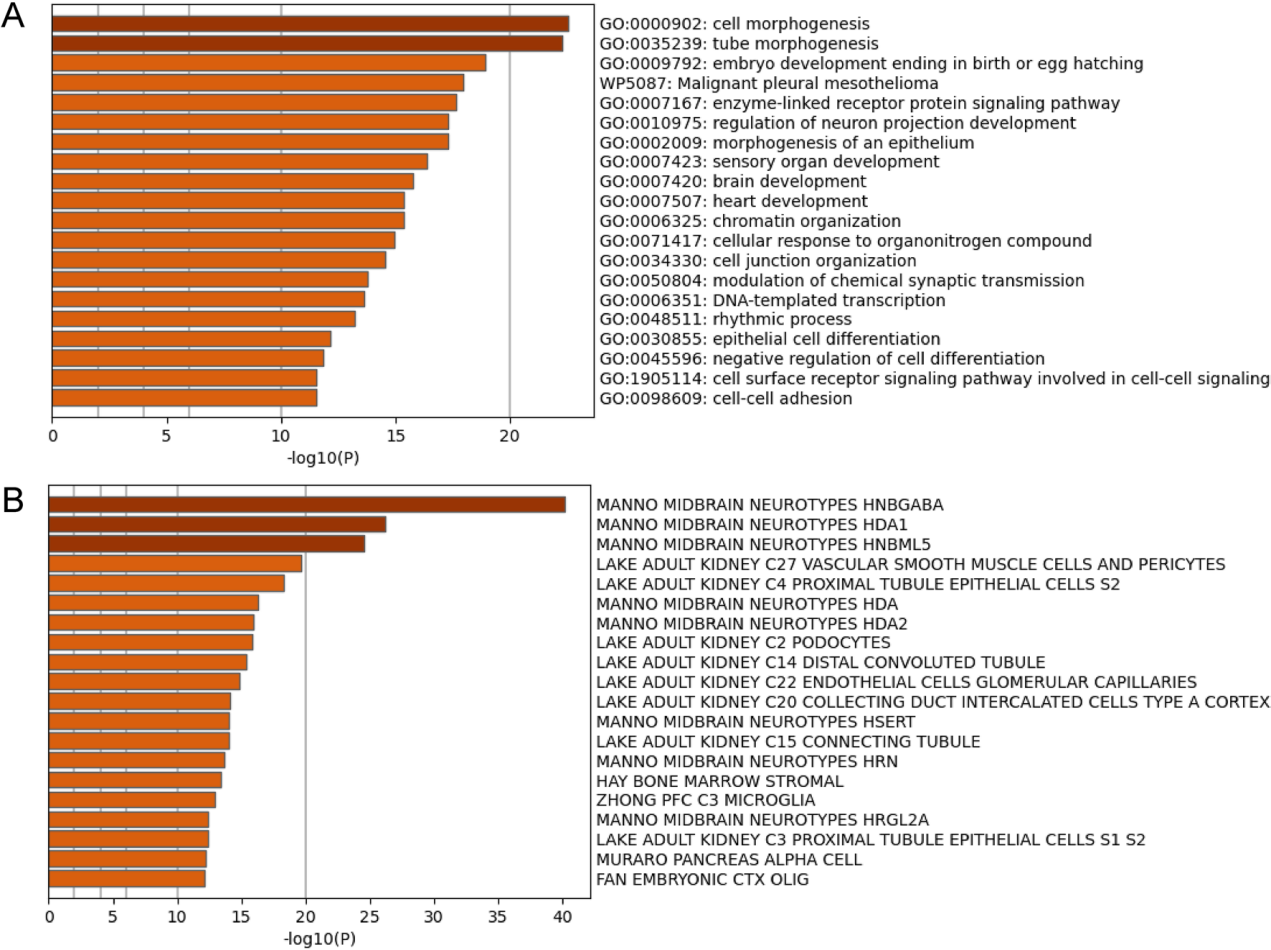
Functional enrichment pathway analysis of the top-50 significantly differentially expressed miRNA-associated genes. The top-20 gene ontology (GO) gene set clusters are shown in the bar graph colored by p-values (**A**). The top-20 cell-type signature gene set clusters are shown in the bar graph colored by p-value (**B**).

The target genes of the top-50 differentially expressed miRNAs were predicted using the miRDB database. Genes with a target score ≥ 90 were subjected to gene set enrichment analysis. Intriguingly, gene ontology (GO) enrichment analysis revealed that pathways related to brain development were linked to the top-20 upregulated pathways (Fig. 5A). Moreover, genes related to fetal midbrain cells were linked to the top-20 genes by cell-type signatures (Fig. 5B).

### miRNA probe PCR assays

**Figure 6:**
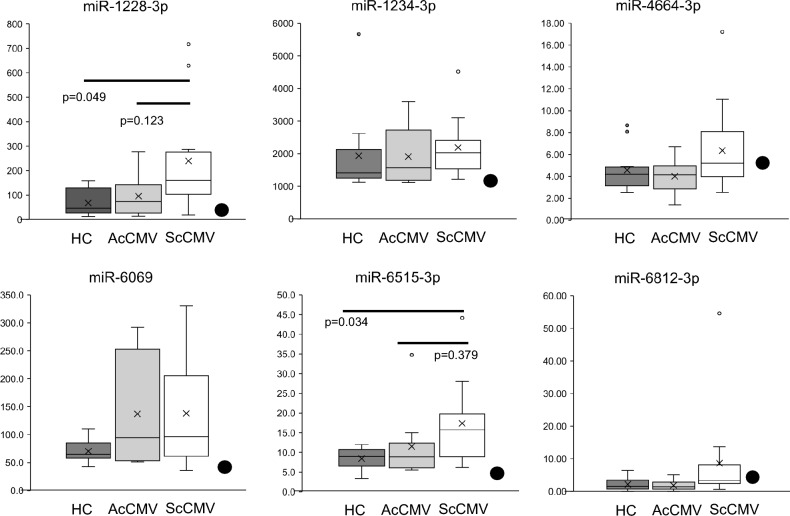
Box-plot diagrams of miRNA expression level analysis via digital PCR. Droplet digital PCR assays were performed to detect hsa-miR-1228-3p, hsa-miR-1234-3p, hsa-miR-4664-3p, hsa-miR-6069, hsa-miR-6515-3p, and hsa-miR-6812-3p levels using a miRCURY LNA miRNA PCR Assay. HC, healthy control; ScCMV, symptomatic cCMV; AScCMV, asymptomatic cCMV. Closed dots indicate the level of late-onset ScCMV.

**Figure 7:**
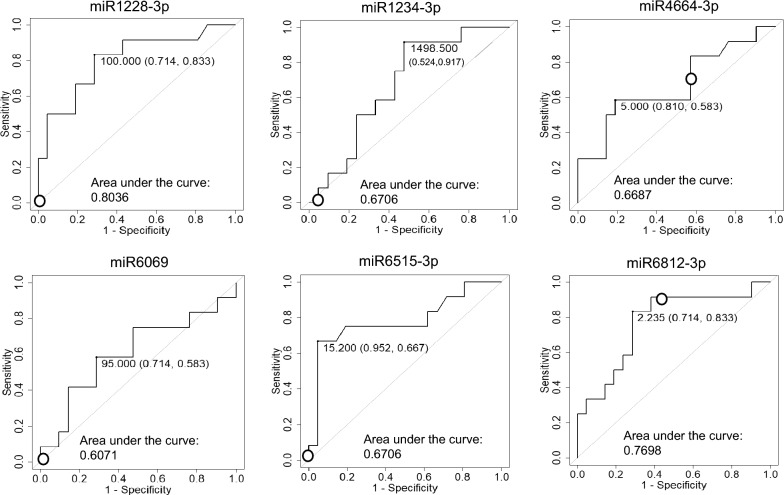
Receiver operating characteristic curve for miRNA expression analysis via digital PCR. A comparative analysis t-test was performed using the R software. Closed dots, cut-off point; open dots, level of a case with late-onset ScCMV.

Six miRNAs (miR-1228-3p, miR-1234-3p, miR-4664-3p, miR-6069, miR-6515-3p, miR-6812-3p) were selected as candidate biomarkers to distinguish not only ScCMV from HC, but also ScCMV from AcCMV. The six miRNAs are selected according to the following requirements: (1) the miRNA significantly differed with corrected p-value < 0.05 (ScCMV vs. HC), and (2) the miRNA differed with a p-value < 0.05 (ScCMV vs. AcCMV). These miRNAs were analyzed using the miRNA probe assay in the digital PCR cohort. The miR-1228-3p and miR-6515-3p levels were significantly higher in patients with ScCMV than in HC (Fig. 6). Receiver operating characteristic (ROC) curve analysis was performed for these miRNAs (Fig. 7). The values of late-onset ScCMV are shown in Fig. 7. Only two miRNAs were above the cut-off point in patients with late-onset ScCMV.

Next, the same analyses were performed with late-onset ScCMV included in the ScCMV group to further compare the groups with neurological disorders and AcCMV and HC. The six miRNAs miR-1228-3p, miR-1234-3p, miR-4664-3p, miR-6069, miR-6515-3p, and miR-6812-3p were selected as the biomarker candidates as well, and miR-1228-3p and miR-6515-3p levels were significantly higher in patients with ScCMV/late-onset ScCMV in the digital PCR analysis (Supplementary Figure S3). ROC curve analysis was also performed for these miRNAs (Supplementary Figure S4).

## Discussion

Patients with ScCMV may be underdiagnosed because a confirmative diagnosis of cCMV should be made within three weeks of age^13^. If universal screening for cCMV becomes a common practice, more babies with cCMV will be diagnosed. It is important to determine whether all babies with cCMV with no or subtle symptoms at birth should be treated to improve their prognosis. The informal International Congenital Cytomegalovirus Recommendations Group provided treatment recommendations and recommended not to routinely treat patients with mild symptoms or asymptomatic patients with isolated SNHL based on insufficient evidence^14^. However, no solution exists for infants who develop neurological impairments months or years after birth. Therefore, a biomarker predicting late-onset sequelae is desirable to expand the treatment of cCMV.

As exosomes can penetrate the blood–brain barrier and circulate throughout the body, serum exosomes may be ideal markers for neuronal diseases. There have been many reports on serum exosomes^7,10^; however, research on urinary exosomes for neuronal diseases is limited^11,12,15^. Tomita et al.^12^ identified exosomal miRNAs in urine as risk factors for psychiatric disorders. They discovered that the expressions of six miRNAs (hsa-miR-486-5p, hsa-miR-199a-3p, hsa-miR-144-5p, hsa-miR-451a, hsa-miR-143-3p, and hsa-miR-142-3p) were elevated in the persistent psychotic-like experience group compared to the remitted group^12^. These results suggest that urine exosomal miRNAs are potential biomarkers for neurological disorders.

Before investigating exosomal miRNAs, we confirmed that L1CAM was present in urine exosomes extracted using our method. L1CAM is a neuronal exosome marker protein^16^. Pathway analysis in the present study revealed the enrichment of brain development pathways and fetal brain cell types. This result suggests that neuronal exosomes are present in urine. L1CAM is also expressed in the kidney^17^. The kidney-related gene set was enriched in the target mRNAs predicted by the significantly differentially expressed miRNAs in ScCMV. The secretion of L1CAM-positive exosomes from the kidney may have been upregulated in ScCMV because CMV replicates in kidney epithelial cells^18^. Since the exosome extraction method used in this study was precipitation-based, exosomes from other organs were also included. Neuronal exosomes can be concentrated using polymer precipitation- and immunoaffinity-based capture methods^19^. This method enables a deeper exploration of neuronal exosomes.

Hierarchical clustering of the microarray data revealed differences in the expression profiles of infants with ScCMV and others (AScCMV and HC). Interestingly, infants with late-onset ScCMV were also grouped into the ScCMV cluster. This finding suggests that neuronal damage not yet detected via neuroimaging can be detected using the miRNA profile. The target mRNAs of the top-50 significantly differentially expressed miRNAs (ScCMV vs. HC) were predicted using the miRDB database. Moreover, pathway enrichment analysis showed the enrichment of pathways related to brain development. In the pathway enrichment analysis of cell signatures, the upregulated gene sets were registered in human embryo midbrain cells^20^.

Changes in miRNA expression may reflect impaired neurodevelopment. Recently, Jiang et al.^21^ characterized cellular and viral miRNA expression during CMV infection in neural progenitor cells. In their study, of the seven differentially expressed cellular miRNAs, miR-1246 was upregulated in CMV-infected cells; miR-1246 was also upregulated in the ScCMV group (FDR-adjusted p = 0.01, fold-change = 10.96) and included in the top-50 significantly differentially expressed miRNAs in our study.

Previous studies have reported the risk factors for severe sequelae of cCMV. The blood, CSF, or urine CMV viral load in infants has been examined in several studies; however, the prognostic value of the viral load is inconclusive^22–25^. Few studies have identified biomarkers that predict late-onset sequelae. Ouellete et al.^26^ investigated the blood transcriptional profiles of patients with cCMV and determined the expression profiles of cCMV in late-onset SNHL. To identify effective biomarkers for discriminating ScCMV (including late-onset ScCMV) in infants, six miRNAs that were significantly differentially expressed in ScCMV in comparison with other groups were selected. To confirm their utility, these six miRNAs were further validated in another cohort using digital PCR. Only two miRNAs (miR1228-3p and miR6515-3p) were significantly upregulated in patients with ScCMV compared to HC, although there were no reports suggesting the relationship between these 6 miRNAs and neurodevelopment or hearing. This results could not be replicated using digital PCR. The discrepancy may have been caused by the different data processing procedures of the two methods. The titer of the late-onset ScCMV cases did not reach the cut-off level based on the ROC curve for four of the six miRNAs. Considering that a small number of miRNAs is insufficient for predicting neurological damage, a set of miRNAs can be used for prediction.

This study has several limitations. This is an exploratory study with a relatively small cohort size. Only one patient was diagnosed with late-onset ScCMV infection. The AScCMV defined in this study was assessed at least at three years of age; however, a few patients may become symptomatic after assessment. CSF is suitable for investigating neurological disorders, but exosomes in the CSF were not investigated in this study because only a few infants underwent CSF testing via CMV PCR.

In conclusion, the urinary exosomal miRNA profile revealed distinct features of infants with ScCMV infection. Pathway enrichment analysis of predicted target genes using significantly differentially expressed miRNAs suggested an impairment in neurodevelopment. The findings of this study are useful for evaluating neurological damage caused by cCMV. However, further studies are required to validate the identified miRNA candidates found in this study.

## Methods

### Patients and controls

Patients diagnosed with cCMV between March 2012 and November 2019 were recruited at Nagoya University. All patients underwent physical and laboratory examinations, neuroimaging, auditory brainstem response tests, and MRI. The diagnosis of symptomatic cCMV was made based on the criteria defined in a previous study^3^, although CSF was not routinely investigated. Infants were diagnosed with ScCMV if they had any of the following conditions: thrombocytopenia, petechiae, hepatomegaly, splenomegaly, intrauterine growth restriction, hepatitis, or central nervous system involvement such as microcephaly, intracranial calcifications, abnormal CSF indices, chorioretinitis, and SNHL.

Patients with ScCMV are indicated for oral valganciclovir treatment. If babies had no findings, they were diagnosed with AScCMV and followed up without antiviral treatment. Both patients with ScCMV and AScCMV were followed up for auditory and neurodevelopmental assessments up to 3 years of age.

All study protocols were approved by the Institutional Review Board of the Nagoya University Graduate School of Medicine (approval number: 2017-0404-2). Written informed consent was obtained from the parents or legal guardians of all participants. All the experiments were performed in accordance with the Declaration of Helsinki.

### Sample collection

Urine samples were collected at the first visit and stored at -80 ℃ until used for the assays. As controls, urine samples were collected from babies whose mothers were suspected of having CMV infection during pregnancy. All infants in the control group tested negative for CMV and had no clinical findings.

Urine samples from two babies with ScCMV under oral valganciclovir treatment with negative results for urine CMV PCR during sample collection were used for western blotting. The exosomes were derived directly after collection or after preservation for 48 h at -80 ℃. The CSF collected from a patient with Group B streptococcal meningitis was used as a positive control for L1CAM.

### Exosome and exosomal RNA extractions

Urine exosomes were extracted from 1.5-mL of urine using an Exo-urine EV Isolation kit (System Biosciences, Palo Alto, CA, USA) according to the manufacturer’s instructions. CSF exosomes were extracted from 1000 µL of CSF using a miRCURY Exosome Cell/Urine/CSF kit (Qiagen, Hilden, Germany). The miRNAs were extracted from urine exosomes using an EVery EV RNA Isolation Kit (System Biosciences) according to the manufacturer’s instructions.

### Western blot

Urine and CSF exosomes were analyzed for CD9 and L1CAM expression using western blotting. Exosomes were lysed in Laemmli sample buffer (Bio-Rad, Hercules, CA, USA) containing β-mercaptoethanol (Katayama Chemical Industries, Osaka, Japan). Proteins were separated using SDS-PAGE on a 4–15% polyacrylamide gel and transferred onto a nitrocellulose membrane. The membranes were pretreated in Tris-buffered saline-Tween 20 with 5% dry milk for 1 h and then incubated overnight at 4 °C with primary antibodies (anti-CD9 clone 12A12, Cosmo Bio, Tokyo, Japan; anti-L1CAM, clone UJ127, Abcam, Cambridge, UK). The membranes were washed three times with Tris-buffered saline-Tween 20 and incubated with the corresponding secondary antibodies (horseradish peroxidase-linked anti-mouse IgG, Cell Signaling Technology, Danvers, MA, USA) for 1 h at room temperature.

### miRNA microarray analysis

The expression levels of 2588 miRNAs were studied using a SurePrint G3 Human miRNA microarray 8 × 60K (Agilent Technologies, Santa Clara, CA, USA). One nanogram of RNA from each sample was used in the assay. The experiment was performed by DNA Chip Research, Inc. (Tokyo, Japan).

### miRNA probe droplet digital PCR assays

A droplet digital PCR assay with Evagreen (Bio-Rad) was performed to detect the expressions of hsa-miR-1228-3p, hsa-miR-1234-3p, hsa-miR-4664-3p, hsa-miR-6069, hsa-miR-6515-3p, and hsa-miR-6812-3p using a miRCURY LNA miRNA PCR Assay (Exiqon, Vedbaek, Denmark). Five hundred picograms of RNA were used in each assay. The experiment was performed by DNA Chip Research, Inc.

### Data analysis

For microarray analysis, the intensity of each hybridization signal was evaluated using the Extraction Software Version A.7.5.1 (Agilent Technologies, Santa Clara, CA, USA). Principal component, hierarchical clustering, and differential gene expression analyses were performed using the GeneSpring software (Agilent Technologies) and the R software limma package^27^. Comparative analysis was performed using fold-changes and an independent t-test. The false discovery rate was controlled by adjusting the p-value using the Benjamini–Hochberg algorithm. The target genes of the miRNAs were selected using the mirDB database^28,29^. Gene set enrichment analysis was performed using the Metascape software^30^. For PCR analysis, a comparative t-test was performed using the R software. ROC curves were calculated using the R software pROC package^31^.

### Supplementary Information

Supplementary Information 1.

Supplementary Information 2.

## Data Availability

The datasets generated and analyzed during the current study are available in the Gene Expression Omnibus repository under the accession number GSE252811.
