# Supplementary material for: MicroRNA expression profiling of urine exosomes in children with congenital cytomegalovirus infection
Source: Sci Rep. 2024 Mar 5;14:5475. doi: 10.1038/s41598-024-56106-1 (PMC10914720; doi:10.1038/s41598-024-56106-1)
Supplement: Supplementary file 2 — Supplementary Information 2. [file 41598_2024_56106_MOESM2_ESM.pdf]

Supplementary Information for:

MicroRNA expression profiling of urine exosomes in children with congenital cytomegalovirus infection

Yuka Torii[1], Takako Suzuki[1], Yuto Fukuda[1], Kazunori Haruta[1], Makoto Yamaguchi[1], Kazuhiro Horiba[2], Jun-ichi Kawada[1], Yoshinori Ito[3]

[1]Department of Pediatrics, Nagoya University Graduate School of Medicine, 65 Tsurumai-cho, Showa-ku, Nagoya 466-8550, Japan

[2] Pathogen Genomics Center, National Institute of Infectious Diseases, Toyama 1-23-1, Shinjuku-ku, Tokyo 162-8640, Japan

[3] Department of Pediatrics, Aichi Medical University, Nagakute, 480-1195, Japan

*Corresponding author: Yoshinori Ito, M.D., Ph.D.

Department of Pediatrics, Aichi Medical University

1-1 Yazakokarimata, Nagakute, 480-1195, Japan

Tel: +81-561-62-3311, Fax: +81-561-63-4835

E-mail: yoshi-i@med.nagoya-u.ac.jp

This file contains:

Supplementary Figures S1-S4

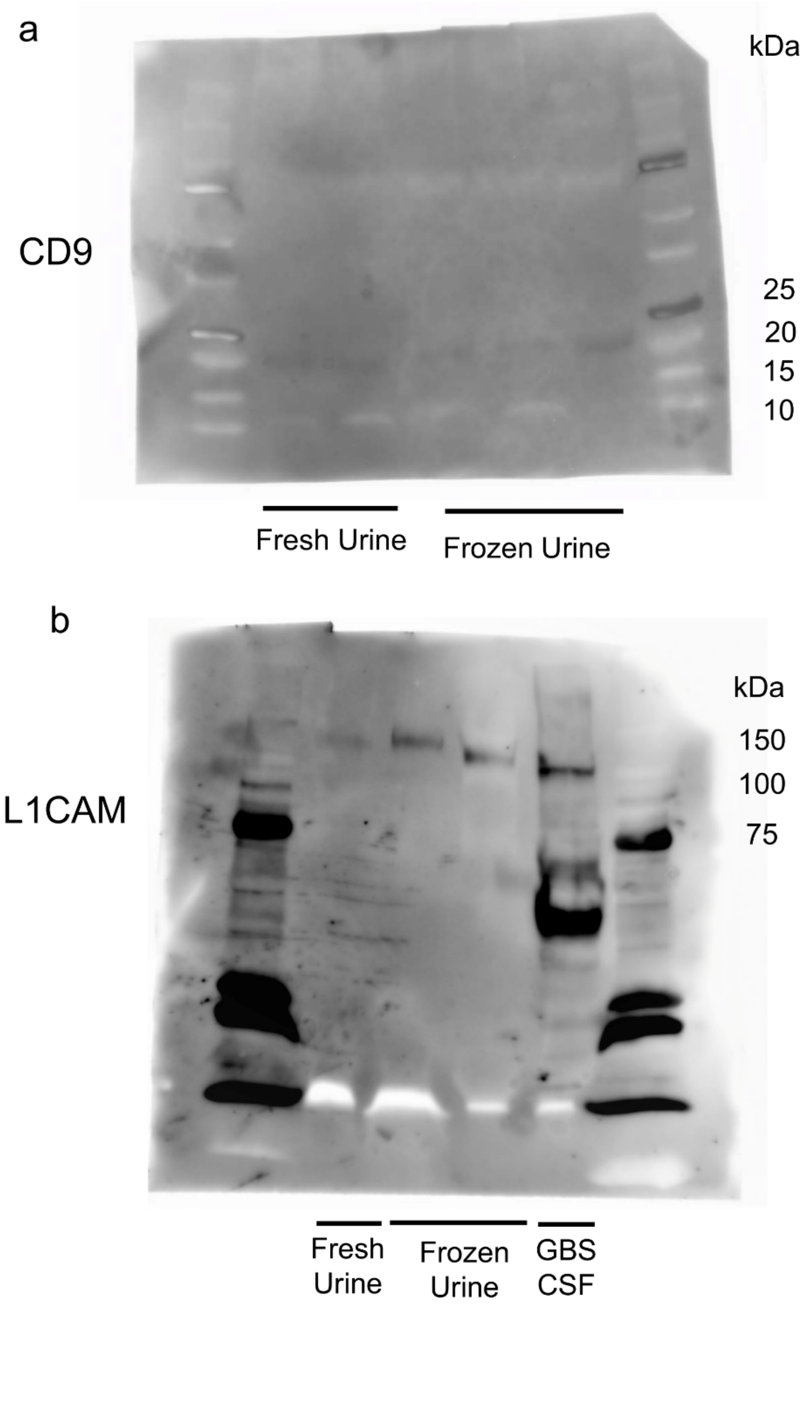

Supplementary figure S1. The uncropped images of all Western blots. Expression of CD9 in exosomes obtained from frozen and fresh urine samples (a). Expression of L1CAM in exosomes from frozen urine, fresh urine (cCMV), and the cerebrospinal fluid of patients with Group B streptococcus infection (b).

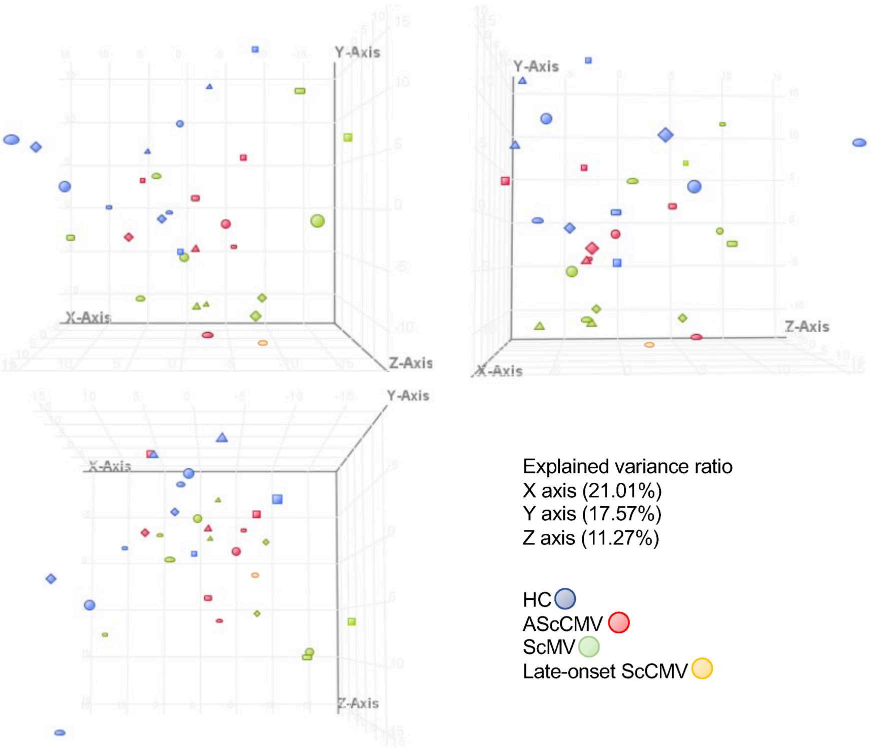

Figure S2

Supplementary figure S2. Three-dimensional principal component analysis of miRNA microarray data. Three-dimensional principal component analysis was performed using the GeneSpringand R software limma packages. The four groups (ScCMV, late-onset ScCMV, AScCMV, and HC) are indicated in different colors. scCMV, symptomatic congenital cytomegalovirus (CMV) infection; AScCMV, asymptomatic CMV infection; HC, healthy controls.

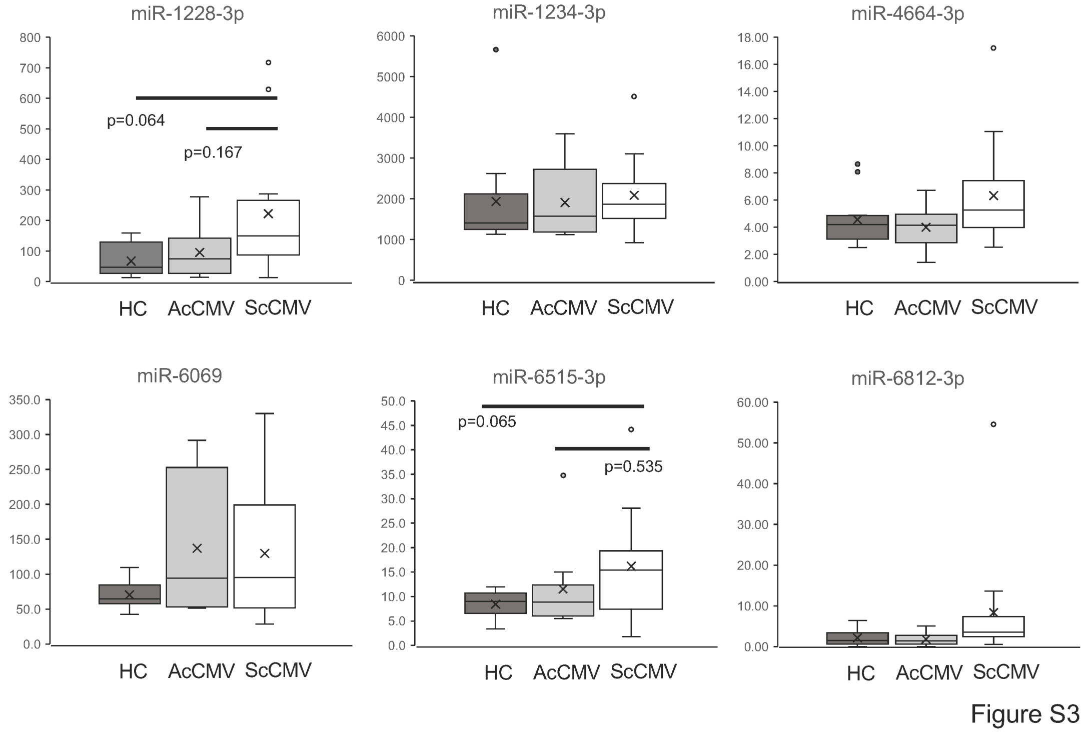

Supplementary Figures S3. Box-plot diagrams of miRNA expression level analysis via digital PCR (further analysis including a late-onset ScCMV case in the ScCMV group). Droplet digital PCR assays were performed to detect hsa-miR-1228-3p, hsa-miR-1234-3p, hsa-miR-4664-3p, hsa-miR-6069, hsa-miR-6515-3p, and hsa-miR-6812-3p levels using a miRCURY LNA miRNA PCR Assay. HC, healthy control; ScCMV, symptomatic cCMV; AScCMV, asymptomatic cCMV.

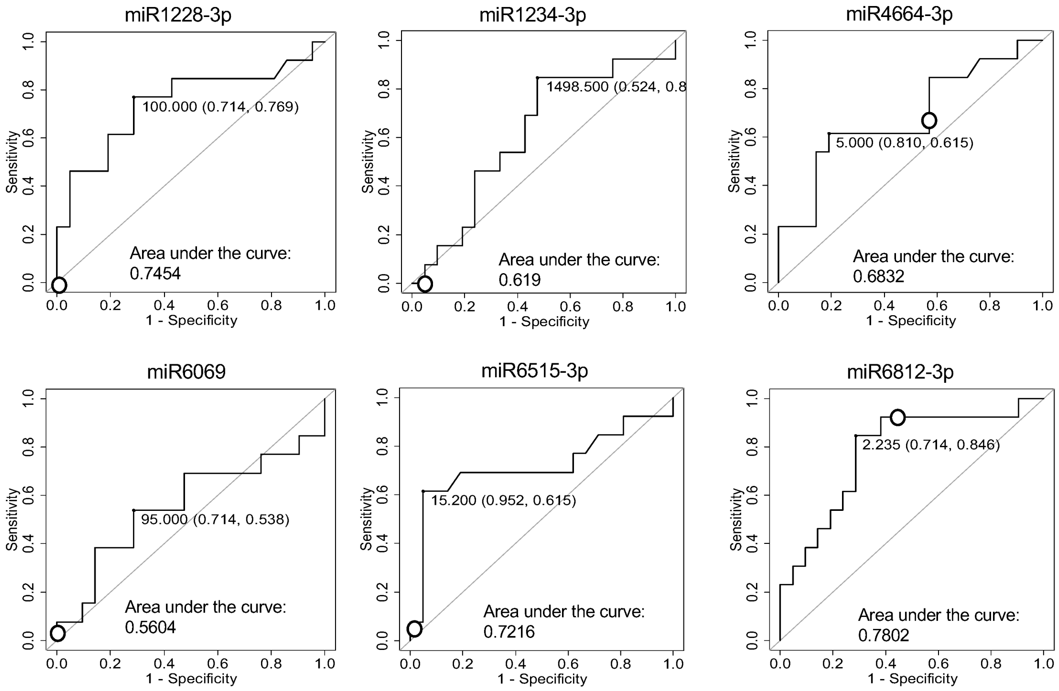

Figure S4

Supplementary Figures S4. Receiver operating characteristic curve for miRNA expression analysis via digital PCR (further analysis including a late-onset ScCMV case in the ScCMV group). A comparative analysis t-test was performed using R software. Closed dots, cut-off point; open dots, level of a case with late-onset ScCMV.
